# ID 147 - Revisão Sistemática de Ensaios Clínicos Randomizados Aponta para Incertezas sobre os Benefícios e os Riscos do Método ABA para Transtorno do Espectro Autista

**DOI:** 10.5327/2237-9622.2025.v34s1.147

**Published:** 2025-11-25

**Authors:** Roberta Borges Silva, Ana Luiza Cabrera Martimbianco, Camila Monteiro Cruz, Carolina de Oliveira Cruz Latorraca, Cecília Menezes Farinasso, Isabela Porto de Toledo, Patrícia do Carmo Silva Parreira, Rafael Leite Pacheco, Roberta Borges Silva, Verônica Colpani, Rachel Riera

**Keywords:** transtorno do espectro autista, TEA, análise do comportamento aplicada, ABA, revisão sistemática

## Abstract

**Introdução:**

A prevalência de Transtorno do Espectro Autista (TEA) no Brasil e no mundo é estimada em 1% e dados do Sistema Único de Saúde (SUS) apontam que essas pessoas realizaram 9,6 milhões de atendimentos ambulatoriais em 2021. O método Applied Behavior Analysis (ABA) é uma terapia comportamental estruturada, intensiva e precoce que visa promover independência, aumentar a funcionalidade e a qualidade de vida de pessoas com TEA por meio de mudanças de comportamento, utilizando os princípios da teoria da aprendizagem, como reforço positivo. O objetivo deste estudo foi avaliar os efeitos do método ABA para pessoas com TEA.

**Método:**

Revisão sistemática, conduzida no Nats/NEv do Hospital Sírio-Libanês, seguindo as recomendações metodológicas do Cochrane Handbook for Systematic Reviews of Interventions e relatada de acordo com o Preferred Reporting Items for Systematic Reviews and Meta-Analyses (PRISMA 2020). O protocolo foi registrado prospectivamente na base PROSPERO (https://www.crd.york.ac.uk/prospero/display_record.php?ID=CRD42024499388). Em 8/1/2024, foi realizada busca ampla e sensível em bases eletrônicas (ADOLEC, CENTRAL, Embase, LILACS, MEDLINE, PsycNET), literatura cinzenta (DANS), registros de ensaios clínicos (clinicaltrials.gov e ICTRP-WHO) e busca manual em listas de referências. Foram considerados ensaios clínicos randomizados (ECR) que avaliaram o método ABA (original ou adaptado) para pessoas com TEA comparado com nenhuma intervenção, lista de espera, outras psicoterapias ou terapias múltiplas. Desfechos primários: melhora global e gravidade dos sintomas; secundários: interação social, comportamento adaptativo e social, comunicação verbal e não verbal, qualidade de vida, habilidade cognitiva, estereotipia, satisfação e avaliação dos cuidadores e eventos adversos. O risco de viés dos ECR foi avaliado com a tabela de risco de viés da Cochrane e a certeza da evidência foi avaliada pela abordagem GRADE

**Resultados:**

Foram identificadas 7.689 referências e, após o processo de seleção, 11 ECR (287 participantes) foram incluídos (oito com resultados disponíveis e três em andamento). Os principais desfechos avaliados foram interação social, comunicação verbal e não verbal e estereotipias. Considerando todas as comparações, a certeza da evidência para todos os desfechos foi ‘muito baixa’, indicando que há incerteza quanto aos benefícios e segurança do método ABA para TEA. Contribuíram para esta incerteza: a baixa qualidade metodológica (e o alto risco de viés) dos ECR, a heterogeneidade das modalidades de ABA utilizadas, a diversidade dos desfechos avaliados e das ferramentas utilizadas para mensuração, a imprecisão dos resultados numéricos e ao relato insuficiente das informações relevantes.

**Conclusão:**

de acordo com os resultados dos ECR disponíveis até o momento, os benefícios e os riscos do método ABA estruturado para o tratamento de pessoas com TEA, quando comparado a nenhum tratamento, lista de espera, outras psicoterapias ou terapias múltiplas são incertos. Diante desta incerteza, é importante discutir a prevalente indicação deste método na prática, considerando aspectos como heterogeneidade de sua aplicação, capacidade instalada, disponibilidade de profissionais capacitados para sua correta aplicação no sistema público e na saúde suplementar, existência de terapias não farmacológicas para compor o cuidado oferecido e desconhecimento sobre os efeitos clínicos do método ABA também no longo prazo.

